# Plastics and Plastic Fillings

**Published:** 1881-05

**Authors:** 


### BIBLIOGRAPHICAL.

*Plastics and Plastic Filling.* By J. Foster Flagg, D. D. S. Publisher: Presley Blackiston, Philadelphia, 1881.

At length we have the results of what has been termed the "New Departure" compiled in the form of a volume of some two hundred pages, gotten up in a manner which is a credit to both author and publisher. The articles composing this work were prepared for magazine use, we are informed in the preface, and while many may not coincide with the extreme views of the author on plastics, yet all must confess that a great deal of very useful information can be found in this volume.

It is dedicated to the "New Departure Corps," as a testimonial of individual indebtedness, sincere respect and kind regard with a monogram, the motto of which is "Truth, without Fear and without Favor." All varieties of plastic materials are described, with the mode of manipulation: criticisms on papers of other practitioners; attributes of certain metals, and general considerations concerning amalgams, such as their local and systemic effects, galvanic electricity, irritation, salivation, bad taste, pulp devitalization, periodontitis, alveolar abscess, exostosis, necrosis; all of which effects the author regards as impossibilities at the hands of skilful operators, or in other words, from the use of proper material in a proper manner. The author asserts that his principal object is to force all ordinary material from the market, unless they are offered as such. Some of the unpleasant effects of amalgams, he claims, can be brought about by the presence of gold, and when amalgam is at fault, he obviates this by a resort to gutta-percha, oxychloride and phosphates of zinc with the requisite renewals.

A great amount of useful information can be derived from the articles on gutta-percha, oxychloride of zinc, and phosphate of zinc.

The last article on "Technicalities," relates to what is termed "ageing," "bulging," "buffering," "creviceing," "domeing," "facing," "frotting," "wafering," "trunnioning," &c. The work is illustrated with twelve wood cuts, and is written in the energetic, forcible and self-reliant style peculiar to its author, who, if he cannot convert others to his opinions and modes of manipulation, is honest in promulgating what *he* believes to be true theory.

